# A Novel BODIPY-Zn Complex as Innovative Sonosensitizer for Enhanced Sonodynamic Therapy

**DOI:** 10.3390/molecules30071587

**Published:** 2025-04-02

**Authors:** Jungmin Lee, Soeun Lee, Gihoon Jo, Eunbin Hwang, Junhyoung Lee, Jiyou Han, Hyo Sung Jung

**Affiliations:** 1 Department of Biomedical & Chemical Sciences, Hyupsung University, Hwasung-si 18330, Republic of Korea; rhscndzld56@naver.com (J.L.); thishand01@naver.com (S.L.); whrlgns0601@gmail.com (G.J.); hb4250@naver.com (E.H.); wnsgud101@naver.com (J.L.); 2 Department of Gerontology (AgeTech-Service Convergence Major), Graduate School of East-West Medical Science, Kyung Hee University, Yongin-si 17104, Republic of Korea

**Keywords:** sonodynamic therapy, sonosensitizer, BODIPY-Zn complex, reactive oxygen species, ultrasound

## Abstract

Ultrasound (US)-based sonodynamic therapy (SDT) presents a promising and secure approach to treating cancer with the advantage of enhanced tissue penetration, making it a favorable option compared with traditional photodynamic therapy. However, the search for innovative sonosensitizers that exhibit both high sonosensitizing efficacy and good biocompatibility poses a formidable challenge. In this research, we prepared a novel BODIPY-Zn complex (**BSS-Zn**) incorporating a hydrophilic short polyethylene glycol unit and explored its feasibility as a sonosensitizer. **BSS-Zn** exhibited enhanced reactive oxygen species (ROS) generation behavior upon US irradiation, outperforming a control sensitizer, **BSS** (an analog lacking the Zn complex), and a commercial sonosensitizer, ZnPc (currently undergoing clinical testing), with regard to sonosensitizing properties. The enhanced effect of **BSS-Zn** was attributed to increased levels of ROS, such as hydroxyl radicals, singlet oxygen, and superoxide, mediated by US exposure in aqueous media. The SDT effect of **BSS-Zn** on MDA-MB-231 cells was verified by confirming the intracellular types of generated ROS and evaluating the cytotoxicity to MDA-MB-231 cancer cells. This pioneering study highlights the potential of **BSS-Zn** as an innovative sonosensitizer for SDT. Our findings provide valuable guidance for the design of efficient sonosensitizers.

## 1. Introduction

Sonodynamic therapy (SDT)—an offshoot of photodynamic therapy (PDT)—is rapidly emerging as a noninvasive modality for treating cancer in both fundamental research and clinical practice [1,2]. In SDT, reactive oxygen species (ROS) are generated, and sonocavitation is induced to damage cancer cells and tissues, which is typically achieved through the combination of low-intensity ultrasound (US) irradiation and precisely administered sonosensitizers [3]. Compared with PDT, which relies on light as the energy source and is constrained by the penetration depth, US has the advantage of deeper tumor penetration with minimal effects on adjacent normal tissues. Thus, it is promising for addressing deep-seated tumors and exhibits favorable safety profiles compared with conventional cancer therapies [4].

The overall effectiveness of SDT depends significantly on the sonosensitizer’s performance. To date, SDT has commonly employed various organic sonosensitizers (porphyrins and their derivatives [5,6], phthalocyanines [7,8], xanthenes [9], and other small-molecule agents [10,11]) and inorganic sonosensitizers (zinc oxide nanoparticles [12], black phosphorus [13], iron oxide nanoparticles [14], and metal–organic frameworks [15]). Despite these efforts, the lack of diversity among sonosensitizers and their suboptimal efficacy—specifically their limited ROS generation efficiency, poor bioavailability, and insufficient sonodynamic cytotoxicity—necessitates the development of more effective sonosensitizers, as their current performance has not yet met the standards required for clinical application [16]. Recently, considerable efforts have been directed toward the development of novel sonosensitizers with improved properties to address these issues.

Boron-dipyrromethenes (BODIPYs) have extensive applications in fluorescence imaging and labeling owing to their optical, thermal, photo-, and chemical stability, as well as their high molar absorptivity and minimal biological toxicity [17,18]. To utilize existing BODIPY dyes in PDT, it is essential to implement suitable compound modification strategies. Several approaches, such as introducing halogens into the BODIPY core and creating BODIPY dyads or dimers, have been employed to enhance the efficacy of BODIPY as a triplet photosensitizer [19,20]. This modification allows triplet BODIPY sensitizers to participate in bimolecular sensitization processes, such as the production of singlet oxygen (^1^O_2_), which is utilized in PDT.

The utilization of BODIPY–metal complexes presents an appealing avenue for enhancing outcomes related to photoactivity. These complexes have exhibited significant effects on the generation of ROS, which is attributed to the long-lived BODIPY triplet excited state due to strong spin–orbit coupling between the heavy metal atoms and the BODIPY’s core [21]. A few BODIPY sensitizers, including Cu [22,23], Zr [24], Ru [25], and Pt [26], have been explored for enhancing photosensitizing efficacy.

The selection of the metal ions in a complex is paramount for biological applications. The utilization of 4d/5d transition-metal ions is associated with the risk of heavy metal toxicity—a well-known issue for cisplatin and its analogs, as well as Ir, Rh, and Ru complexes [27,28]. An ideal choice is a bioessential 3d metal ion to mitigate heavy metal toxicity. We opted for 3d^10^-Zn^2+^ over redox-active 3d transition-metal ions such as Cu, Fe, and Mn. The latter metal ions can generate ROS, specifically hydroxyl radicals (•OH), even under dark conditions, which may not be ideal for PDT/SDT. Zn^2+^, which is characterized by its non-redox and diamagnetic properties, does not induce the generation of radical species under dark conditions. Notably, Zn^2+^ is expected to stabilize the triplet excited state of sensitizers such as phthalocyanines or BODIPY through coordination, enhancing intersystem crossing efficiency and consequently, ROS generation under photo-irradiation [29,30]. Nevertheless, to the best of our knowledge, advanced SDT strategies involving BODIPY-Zn that can improve ROS generation have not yet been developed.

Given that the responsiveness of sonosensitizers to sonication is often correlated with their photosensitizing and photocatalytic capabilities, we postulated that the BODIPY-Zn complex could exhibit noteworthy sonosensitizing efficacy [31]. In this study, we synthesized a BODIPY sonosensitizer-Zn complex called **BSS-Zn**, which is a novel type of sonosensitizer incorporating a hydrophilic short polyethylene glycol (PEG) moiety. Based on the ROS production rate and types of ROS under US irradiation, **BSS-Zn** was demonstrated to exhibit excellent sonosensitizing activity compared with reference sensitizers (the Zn-free BODIPY-based sensitizer **BSS** and the well-known sensitizer ZnPc). Additionally, the SDT effect of **BSS-Zn** was verified by confirming the intracellular types of generated ROS (•OH, ^1^O_2_, and O_2_^•−^) and evaluating the cytotoxicity to MDA-MB-231 cells.

## 2. Results and Discussion

### 2.1. Design and Characterization of **BSS** and **BSS-Zn**

To design a putative BODIPY-Zn complex sonosensitizer, we formed a rational Zn-binding site by introducing the 2-picolyl-triazole binding motif into the BODIPY structure. We focused on BODIPY as a sensitizer owing to its optical and chemical stability, high molar absorptivity, high ROS production efficiency, and sensitizing ability [19]. However, because most BODIPYs are hydrophobic, modifying the existing BODIPY with a short PEG linker via the 2-picolyl-triazole unit enhances not only its water solubility but also its biocompatibility [32]. The 2-picolyl-triazole unit forms a stable complex with transition-metal ions, such as Zn^2+^ ions [33].

**Figure 1:**
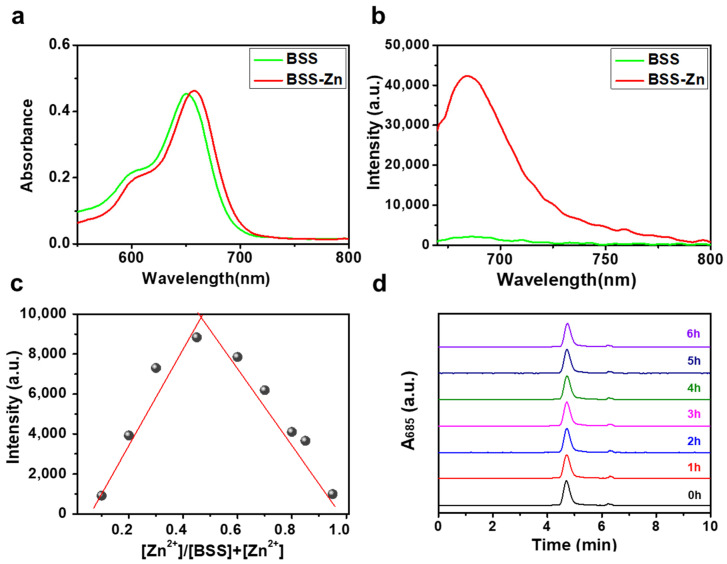
Effect of Zn^2+^ complexation on **BSS**. (**a**) UV-Vis and (**b**) fluorescence spectra of **BSS** and **BSS-Zn** (5.0 μM) in MeCN. (**c**) Job’s plot analysis of solution mixtures of **BSS** and Zn^2+^. (**d**) HPLC chromatogram of **BSS-Zn** in a PBS solution (pH 7.4, 10 mM) over a period of 6 h. Excitation at 660 nm (slit width of 2.5/2.5 nm).

**Scheme 1:**
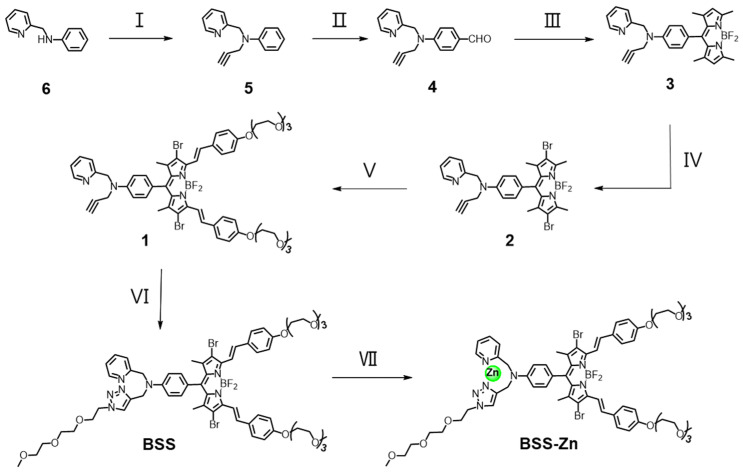
Synthesis pathways of **BSS-Zn** and **BSS**. Reagents and conditions: (I) propargyl bromide, potassium carbonate, DMF, 60 °C, 16 h, 64%; (II) phosphoryl chloride, DMF, 100 °C, 4 h, 83%; (III) trifluoroacetic acid, 2,4-dimethylpyrrole, dichloromethane, room temperature (rt), 8 h; boron trifluoride diethyl etherate, *p*-chloranil, triethylamine, 3 h, 55%; (IV) *N*-bromosuccinimide, ethanol, rt, 1 h, 94%; (V) m-PEG4-benzaldehyde, magnesium perchlorate, piperidine, glacial acetic acid, toluene, reflux, 1 d, 48%; (VI) m-PEG3-amine, CuSO_4_, sodium ascorbate, methanol/*N*,*N*-dimethylformamide, 6 h, 65%; (VII) Zn(ClO_4_)_2_, MeCN, rt, 3 h, 80%.

First, **BSS** was successfully synthesized following the method presented in Scheme 1, and its structural integrity was verified by MALDI-TOF/TOF-MS, as well as ^1^H and ^13^C NMR spectroscopy (Figures S1–S3 and Section 3.2). Meaningful evidence supporting the expectation that **BSS** facilitates Zn^2+^ complexation was obtained from spectroscopic studies. Upon adding 1.0 equiv of Zn^2+^ to **BSS**, a 7 nm redshift was detected in the absorption peak at 651 nm (Figure 1a and Figure S4a). The intensity of the emission peak of **BSS** at 682 nm increased significantly (Figure 1b and Figure S4b), which is attributed to the chelation-enhanced fluorescence effect commonly caused by Zn^2+^ complexation [34]. Following the protocol established by Thordarson [35], the 1:1 binding constant for MeCN was calculated as (6.0 ± 0.1) × 10^−7^ M^−1^. Additionally, Job’s plot analysis of Zn^2+^-treated **BSS** supported the binding stoichiometry (1:1) (Figure 1c). The stability of the Zn^2+^ complex with **BSS** was verified via HPLC (Figure 1d) in a PBS solution (pH 7.4, 10 mM) over 6 h.

### 2.2. Photo- and Sonosensitizing Properties of **BSS-Zn** and **BSS**

When considering potential sonosensitizer candidates, it is noteworthy that both **BSS-Zn** and **BSS** originate from photosensitizers [31]. Therefore, we compared the photosensitizing and sonosensitizing properties of **BSS-Zn** and **BSS**. Initially, we explored the photosensitizing properties of **BSS-Zn** and **BSS**—specifically their capacities to generate singlet oxygen (^1^O_2_) under photoirradiation—using DPBF as the ^1^O_2_ probe (Figure S5) [36]. Exposure of **BSS-Zn** and **BSS** solutions containing DPBF to a 660 nm laser (100 mW/cm^2^) gradually reduced the spectral absorption intensity associated with DPBF (over 15 min). Thus, both **BSS** and **BSS-Zn** exhibited substantial generation of ^1^O_2_, which aligns with the characteristics of existing BODIPY-based photosensitizers [18].

**Figure 2:**
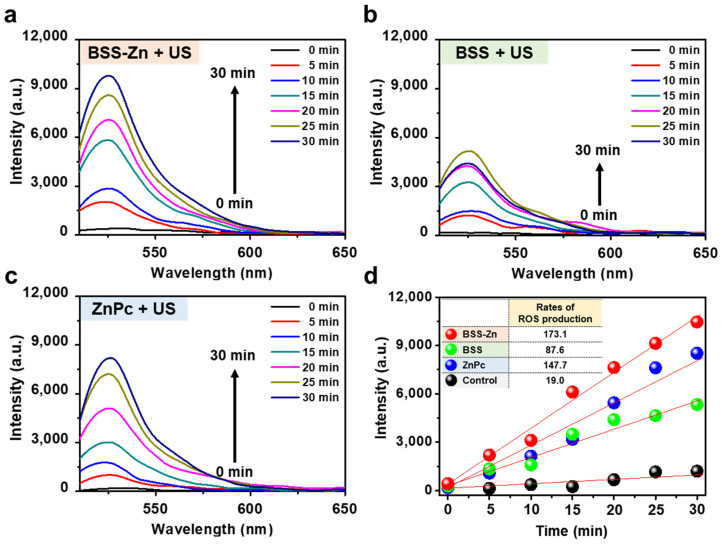
Sonosensitized ROS generation by **BSS-Zn**, **BSS**, and ZnPc. Time-dependent fluorescence changes observed for solutions of 10 μM DCF with 5 μM (**a**) **BSS-Zn**, (**b**) **BSS**, or (**c**) ZnPc under US exposure (0.5 W/cm^2^, 20% duty cycle, 1 MHz) with excitation at 500 nm (slit width of 1/2.5 nm). (**d**) Plots of the signal change in the fluorescence at 525 nm for the experiments shown in (**a**–**c**). The rate of ROS production is represented by the slope of each item shown in the graph.

Recent findings have revealed that certain important transition-metal complexes can function as sonosensitizers capable of generating ROS under US irradiation [37]. To verify the potential of the BODIPY-Zn complex **BSS-Zn** for enhancing sonosensitizing properties under US irradiation, the production of ROS by **BSS-Zn** and the Zn-free **BSS** was quantified using a representative ROS indicator, i.e., DCF [38]. As shown in Figure 2, under US irradiation (0.5 W/cm^2^, 20% duty cycle, 1 MHz), the most significant fluorescence changes in DCF were observed for the **BSS-Zn** group containing the Zn^2+^ ion. The **BSS-Zn** group exhibited an ROS generation rate approximately 1.98 times higher than that of the **BSS** group. Furthermore, in a comparative analysis with the well-established sensitizer ZnPc [39], which is currently undergoing phase 2 clinical trials, **BSS-Zn** generated ROS > 1.17 times faster under identical experimental conditions (Figure 2). In contrast, no emission change in DCF was observed for solutions containing only DCF (negative control) or Zn^2+^ ions (as the perchlorate salt) under identical experimental conditions (Figure S6). These results suggest that BODIPY sensitizers, which are primarily PDT sensitizers, can also be used as sonosensitizers. In particular, the BODIPY-Zn complex **BSS-Zn** exhibited excellent sonosensitizing properties, indicating its potential for SDT.

### 2.3. Three Different Types of ROS Generation Assays Using US-Irradiated **BSS-Zn** and **BSS**

**Figure 3:**
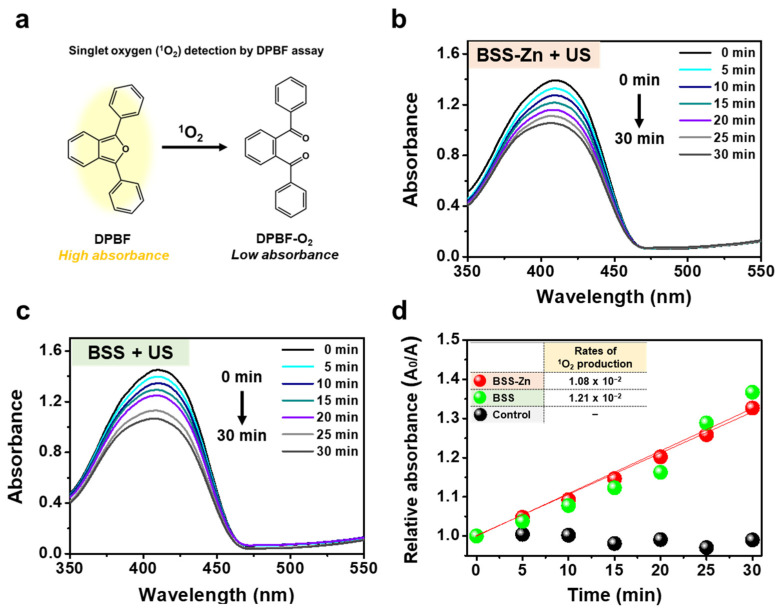
^1^O_2_ generation assay for US-irradiated **BSS-Zn** and **BSS**. (**a**) Illustration of the suggested action mechanism of DPBF as a probe for ^1^O_2_. Time-dependent fluorescence changes observed for solutions of 80 μM DPBF with 1 μM (**b**) **BSS-Zn** or (**c**) **BSS** under US exposure (0.5 W/cm^2^, 20% duty cycle, 1 MHz). (**d**) Plots of the signal change in the relative absorbance (A_0_/A) at 412 nm for the experiments presented in (**b**,**c**). The rate of ^1^O_2_ production is represented by the slope of each item shown in the graph.

Subsequently, the sonosensitizing mechanism of **BSS-Zn** was explored by confirming three different types of ROS (i.e., ^1^O_2_, •OH, and O_2_^•−^) commonly generated in SDT [40]. The types of ROS generated by US-irradiated **BSS-Zn** and **BSS** were confirmed using commercial indicators: DPBF for ^1^O_2_, HPF for •OH, and DHR123 for O_2_^•−^ [41]. Under US irradiation (0.5 W/cm^2^, 20% duty cycle, 1 MHz), the absorption signals of DPBF (as a ^1^O_2_ probe, Figure 3a) decreased at similar rates over 30 min for **BSS-Zn** and **BSS**, suggesting comparable generation of ^1^O_2_, as illustrated in Figure 3b,c.

**Figure 4:**
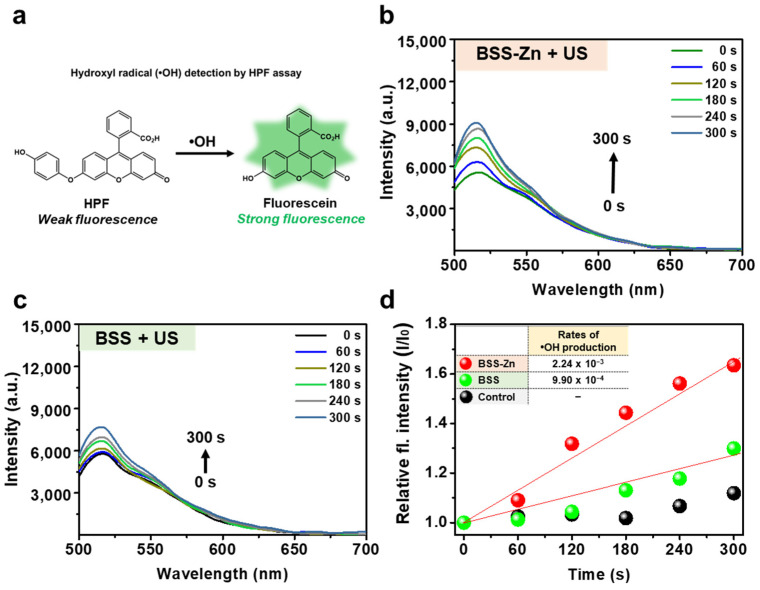
•OH generation assay for US-irradiated **BSS-Zn** and **BSS**. (**a**) Illustration of the suggested action mechanism of HPF as a probe for •OH. Time-dependent fluorescence changes observed for solutions of 10 μM HPF with 5 μM (**b**) **BSS-Zn** or (**c**) **BSS** under US exposure (0.5 W/cm^2^, 20% duty cycle, 1 MHz); irradiation was performed at 490 nm (Xe lamp, slit width of 5/5 nm) in the two experiments. (**d**) Plots of the signal change in the fluorescence at 516 nm for the experiments presented in (**b**,**c**). The rate of •OH production is represented by the slope of each item shown in the graph.

As shown in Figure 4, for the US-irradiated **BSS-Zn** solution containing HPF (as an •OH probe, Figure 4a), the fluorescence intensity of HPF increased significantly over time (300 s), whereas the **BSS** solution exhibited smaller fluorescence increases under the same US irradiation conditions. Under these experimental conditions, the rate of •OH generation in the **BSS-Zn** solution was approximately 2.26 times that in the **BSS** solution (Figure 4d).

**Figure 5:**
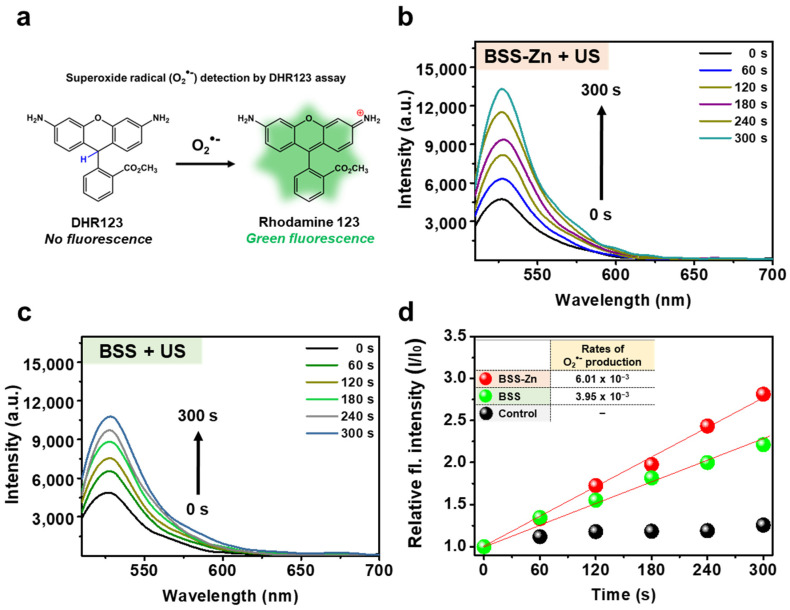
O_2_^•−^ generation assay for US-irradiated **BSS-Zn** and **BSS**. (**a**) Illustration of the suggested action mechanism of DHR123 as a probe for O_2_^•−^. Time-dependent fluorescence changes observed for solutions of 10 μM DHR123 with 10 μM (**a**) **BSS-Zn** or (**b**) **BSS** under US exposure (0.5 W/cm^2^, 20% duty cycle, 1 MHz) with excitation at 500 nm (slit width of 2.5/2.5 nm). (**d**) Plots of the signal change in the fluorescence at 527 nm for the experiments presented in (**b**,**c**). The rate of O_2_^•−^ production is represented by the slope of each item shown in the graph.

US exposure of the **BSS-Zn** solution containing DHR123 (as O_2_^•−^ probe, Figure 5a) significantly increased (by a factor of approximately 1.52) the fluorescence intensity of DHR123 compared with that of the **BSS** solution. In addition, under identical US irradiation conditions, negligible changes were observed in the spectra of the three ROS probes in the absence of **BSS-Zn** or **BSS** (Figures S7–S9). These results indicate that **BSS-Zn** has considerable potential as a sonosensitizer.

### 2.4. In Vitro Investigation of **BSS-Zn** and **BSS** as Potential Sonosensitizers

**Figure 6:**
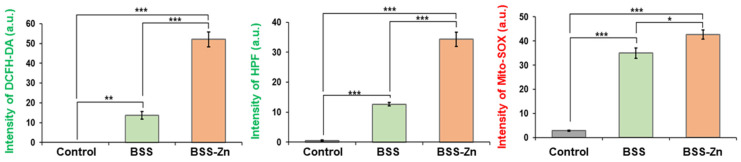
In vitro ROS generation assay for US-irradiated **BSS-Zn** and **BSS**. The fluorescence intensity was measured immediately after the termination of irradiation using 485 nm excitation and 530 nm emission filters for DCFH-DA and HPF and 410 nm excitation and 610 nm emission filters for MitoSOX. * *p* value < 0.05, ** *p* value < 0.01, and *** *p* value < 0.005.

To investigate the potential sonosensitizing effect of **BSS-Zn** in actual biological samples, we assessed intracellular ROS production in MDA-MB-231 cells using the ROS indicator DCFH-DA [38]. After the MDA-MB-231 cells pretreated with **BSS-Zn** or **BSS** (5 μM each) for 24 h were exposed to US irradiation (0.5 W/cm^2^, 20% duty cycle, 1 MHz) for 1 min, a considerable fluorescence enhancement of DCF was observed for the **BSS-Zn** group (Figure 6). However, the **BSS** group exhibited slightly weaker fluorescence. Next, using the same experimental conditions, the generation of •OH and O_2_^•−^ was assessed using HPF and MitoSOX as indicators, respectively [41]. As shown in Figure 6, under US irradiation (1 min, 0.5 W/cm^2^, 20% duty cycle, 1 MHz), the application of **BSS-Zn** to MDA-MB-231 cells containing HPF or MitoSOX significantly increased the fluorescence intensity of HPF or MitoSOX compared with each control. In contrast, the **BSS**-treated group exhibited slightly reduced fluorescence intensities (Figure 6). In addition, no fluorescence of the three ROS indicators was observed in the absence of **BSS-Zn** or **BSS** under identical US irradiation conditions. These results suggest that **BSS-Zn** can play a pivotal role in the generation of ROS in MDA-MB-231 cancer cells exposed to US. Therefore, applying **BSS-Zn** as a sonosensitizer in SDT is expected to result in an enhanced SDT effect, potentially promoting cell death through increased ROS levels within cancer cells.

**Figure 7:**
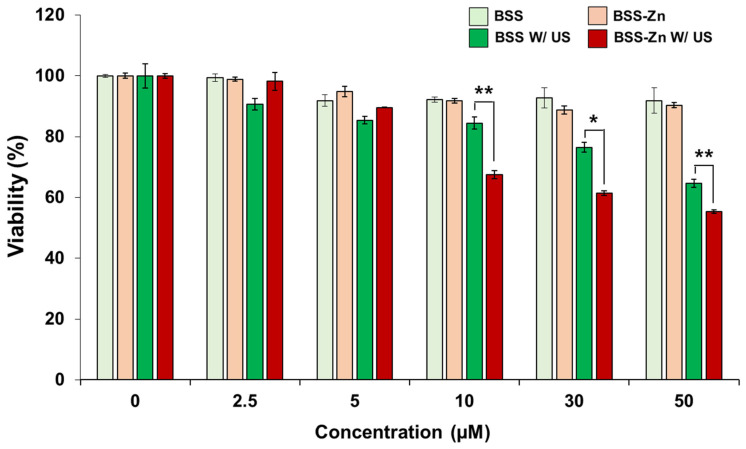
In vitro sonocytotoxicity of **BSS-Zn** or **BSS** and 1% DMSO (as a control) to MDA-MB-231 cells at different concentrations (2.5–50 µM) for 24 h under both US irradiation (1 min, 0.5 W/cm^2^, 20% duty cycle, 1 MHz) and non-irradiation conditions. Statistical significance was determined using a Student’s *t*-test. * *p* value < 0.05 and ** *p* value < 0.01.

To assess the in vitro sonocytotoxicity of **BSS-Zn**, we conducted the WST-8 proliferation assay using MDA-MB-231 cells. Figure 7 depicts the treatment of cells with **BSS-Zn** or **BSS** under both US irradiation (1 min, 0.5 W/cm^2^, 20% duty cycle, 1 MHz) and non-irradiation conditions. The cytotoxicity of the **BSS-Zn** group subjected to US irradiation was notably greater than that of the **BSS** group. The cytotoxic effect of the US-irradiated **BSS-Zn** group on MDA-MB-231 cells showed a dosage-dependent increase. At equal concentration (50 μM), the **BSS-Zn** and **BSS** groups reduced the US-irradiated cell viability by approximately 55% and 65%, respectively (Figure 7). This excellent cytotoxicity may have been due to the enhanced SDT effect resulting from abundant ROS (i.e., ^1^O_2_, •OH, and O_2_^•−^) generated by US-irradiated **BSS-Zn**.

## 3. Materials and Methods

### 3.1. Materials and Instrumentation

All solvents utilized for synthesis and analysis were supplied by Duksan Chemicals (Ansan-si, Republic of Korea) and J.T. Baker Chemicals (Phillipsburg, NJ, USA), respectively. All reagents were purchased from Sigma–Aldrich (St. Louis, MO, USA), except for m-PEG3-amine (AVENTION, Concord, MA, USA), 11-azido-3,6,9-trioxaundecan-1-amine (TCI Chemicals, Tokyo, Japan), 2′,7′-dichlorofluorescin diacetate (DCFH-DA; Invitrogen^TM^), hydroxyphenyl fluorescein (HPF; Invitrogen^TM^, Carlsbad, CA, USA), dihydrorhodamine 123 (DHR123; Invitrogen^TM^), and Mitochondrial Superoxide Indicator Red (MitoSOX; Invitrogen^TM^). Silica gel 60 (Merck, Kenilworth, NJ, USA, 70–230 mesh) served as the stationary phase for both column and thin-layer chromatography (TLC). For TLC, silica gel 60 with a thickness of 0.25 mm was utilized. ^13^C and ^1^H NMR spectra were acquired using 500-MHz NMR spectrometers (Bruker, Billerica, MA, USA), and MALDI-TOF/TOF-MS data were obtained using an autoflex maX instrument (Bruker). An Arc high-performance liquid chromatography (HPLC) system (Waters, Milford, MA, USA), featuring an XBridge^®^ C18 column with dimensions of 4.6 × 250 mm and a particle size of 5 μm, was utilized. Spectroscopic results were acquired using a PerkinElmer Lambda 465 spectrometer (Waltham, MA, USA), and fluorescence spectra were recorded on a Scinco FluoroMate FS-2 spectrofluorophotometer (Seoul, Republic of Korea). US experiments were performed using an Intelect^®^ MOBILE ULTRASOUND system (DJO, LLC, Vista, CA, USA).

### 3.2. Synthesis of **BSS-Zn** and **BSS**

The synthetic pathway of the BODIPY-Zn complex sonosensitizer (**BSS-Zn**) is shown in Scheme 1. All compounds from **6** to **1** were synthesized according to reported procedures [23].

**BSS:** A solution containing compound **1** (0.16 g, 0.14 mmol), m-PEG3-amine (0.022 g, 0.11 mmol), and 10 mol% sodium ascorbate in a solution of methanol/*N*,*N*-dimethylformamide (1 mL/2 mL) was stirred for 30 min at 40 °C. Subsequently, copper(II) sulfate pentahydrate (CuSO_4_·5 H_2_O) (5 mol%) in 0.5 mL of distilled water was introduced into the reaction solution, and stirring was continued for 12 h. The crude mixture obtained by evaporating the solvents under reduced pressure was subjected to purification over silica gel with dichloromethane/methanol (93:7, v/v) as the eluent. This process yielded **BSS** as a dark green solid with a 65% yield (0.12 g): ^1^H NMR (500 MHz, DMSO-d_6_): δ 8.58 (d, J = 4.35 Hz, 1H), 8.06 (s, 1H), 8.03 (d, J = 16.56 Hz, 2H), 7.75 (t, J = 7.63 Hz, 1H), 7.75 (t, J = 7.63 Hz, 1H), 7.59 (d, J = 8.79 Hz, 4H), 7.45 (d, J = 16.56 Hz, 2H), 7.28 (t, J = 7.63 Hz, 1H), 7.21 (d, J = 7.63 Hz, 1H), 7.14 (d, J = 8.61 Hz, 2H), 7.07 (d, J = 8.79 Hz, 4H), 7.01 (d, J = 8.61 Hz, 2H), 4.84 (s, 1H), 4.82 (s, 1H), 4.52 (t, J = 5.83 Hz, 2H), 4.17 (t, J = 4.59 Hz, 4H), 3.84–3.72 (m, 6H), 3.63–3.58 (m, 4H), 3.57–3.49 (m, 10H), 3.48–3.42 (m, 8H), 3.41–3.37 (m, 2H), 3.25 (s, 6H), 3.20 (s, 3H), 1.46 (s, 6H). ^13^C NMR (126 MHz, DMSO-d_6_): δ 160.4, 158.7, 149.8, 149.6, 147.5, 144.3, 141.7, 141.4, 138.7, 137.1, 132.6, 129.6, 129.4, 129.3, 124.1, 122.7, 121.5, 115.8, 115.6, 113.6, 109.8, 71.8, 71.7, 70.5, 70.3, 70.1, 70.1, 70.1, 69.3, 69.3, 67.9, 58.5, 58.5, 56.3, 49.9, 46.7, 32.2, 31.8, 29.5, 14.2. MALDI-TOF/TOF-MS calc. for C_63_H_76_BBr_2_F_2_N_7_O_11_ [M + H]^+^ 1315.40, found 1315.341.

**BSS-Zn:** Zinc perchlorate hexahydrate (Zn(ClO_4_)_2_·6H_2_O) (1 equiv) was gradually added to a stirred solution of **BSS** (0.13 g, 0.10 mmol) in 5 mL of acetonitrile (MeCN). The resulting reaction mixture was stirred at 40 °C for 3 h. After stirring, the solvent was removed from the reaction mixture under reduced pressure. Subsequently, the residue was purified via HPLC using a stationary phase consisting of a C18 column with dimensions of 250 × 4.6 mm and a particle size of 5 μm. The mobile phases utilized were deionized water containing 0.1% trifluoroacetic acid (designated as buffer A) and MeCN (designated as buffer B). Evaporation of the solution produced a dark green powder (0.12 mg, 87% yield). The HPLC chromatogram of **BSS-Zn** is presented in Figure S1, indicating a retention time of 4.70 min.

### 3.3. Photophysical Properties

The photophysical properties of **BSS-Zn** and **BSS** were analyzed by UV-Vis and fluorescence spectroscopy. **BSS**s were prepared using a 0.5 mM dimethyl sulfoxide (DMSO) stock solution, and samples at various concentrations mentioned in the main text were subsequently prepared for analysis. Titration experiments were conducted by fixing the concentration of **BSS** at 5 µM and adding Zn(ClO_4_)_2_ in equivalent amounts. All fluorescence analyses were performed with the slit width set to 2.5/2.5 nm, excitation at 660 nm, and an emission wavelength range of 670–800 nm. Job’s plot experiments for stoichiometry analysis were performed by adjusting the molar ratios of **BSS** and Zn(ClO_4_)_2_ in an MeCN solution.

### 3.4. Assay for Universal ROS

Owing to the self-oxidizing nature of 2′,7′-dichlorodihydrofluorescein (DCF) during long-term storage, the more stable diacetate form—DCFH-DA—was procured from Sigma–Aldrich. To prepare the DCF solution for the analysis of universal ROS generation, DCFH-DA hydrolysis was performed in an NaOH solution. Specifically, 1 mL of 1 mM DCFH-DA in a DMSO solution was combined with 4 mL of a 0.01 M NaOH solution, followed by stirring in the dark for 30 min. The sample (10 µM) was then mixed with DCF (25 µM) in a phosphate-buffered saline (PBS) solution (10 mM, pH 7.4, containing 5% DMSO) and exposed to US (0.5 W/cm^2^, 20% duty cycle, 1 MHz). While the reaction proceeded for 30 min, the fluorescence of the supernatant was measured at 5 min intervals using a Scinco FluoroMate FS-2 spectrofluorophotometer at Ex/Em = 485/535 nm.

### 3.5. Assay for Singlet Oxygen (^1^O_2_) Generation

^1^O_2_ production was assessed by combining 1,3-diphenylisobenzofuran (DPBF) and the sample in an MeCN or PBS solution (10 mM, pH 7.4, containing 50% MeCN) and adjusting the absorption intensities of the sample and DPBF to approximately 0.1 and 1.0, respectively. Following the acquisition of UV-Vis spectra in the absence of light, the samples were exposed to 660 nm light (100 mW/cm^2^) for PDT and US (0.5 W/cm^2^, 20% duty cycle, 1 MHz) for SDT. After each irradiation, UV-Vis spectra covering the range of 300–550 nm were collected, and the absorbance of DPBF was measured at 412 nm over time to establish the slope.

### 3.6. Assay for Hydroxyl Radical (•OH) Generation

To examine •OH generation, the sample (5 µM) was mixed with HPF (10 µM) in a PBS solution (10 mM, pH 7.4, containing 5% DMSO) and exposed to US (0.5 W/cm^2^, 20% duty cycle, 1 MHz). While the reaction proceeded for 5 min, the fluorescence of the supernatant was measured at 30 s intervals using a Scinco FluoroMate FS-2 spectrofluorophotometer at Ex/Em = 485/535 nm.

### 3.7. Assay for Superoxide (O_2_^•−^) Generation

To examine O_2_^•−^ generation, the sample (10 µM) was mixed with DHR123 (10 µM) in a PBS solution (10 mM, pH 7.4, containing 5% DMSO) and exposed to US (0.5 W/cm^2^, 20% duty cycle, 1 MHz). While the reaction proceeded for 5 min, the fluorescence of the supernatant was measured at 30 s intervals using a Scinco FluoroMate FS-2 spectrofluorophotometer at Ex/Em = 500/525 nm.

### 3.8. Assay for Intracellular ROS Generation

After seeding at a density of 2.0 × 10^5^ MDA-MB-231 cells per dish, the cells were incubated for 1 d. Subsequently, the cells were washed twice with PBS before being exposed to a new culture medium, which contained either **BSS-Zn** or **BSS** at a concentration of 5 μM. After a 24 h incubation period, the cells were washed twice with PBS and exposed to 10 μM solutions of DCFH-DA, HPF, or MitoSOX for 30 min. After the PBS washing, US exposure was applied (0.5 W/cm^2^, 20% duty cycle, 1 MHz) for 1 min. The fluorescence intensity was measured immediately after the termination of irradiation using 485 nm excitation and 530 nm emission filters for DCFH-DA and HPF and 410 nm excitation and 610 nm emission filters for MitoSOX.

### 3.9. Assay for In Vitro Cytotoxicity

Cytotoxicity was assessed utilizing WST-8 (WST) obtained from BIOMAX (Guri-si, Gyeonggi-do, Republic of Korea) in accordance with the manufacturer’s instructions. Initially, MDA-MB-231 cells were seeded into a 96-well plate at a density of 1.0 × 10^4^ cells per well. After 1 d, the medium was replaced with 100 μL of fresh medium containing different concentrations (0, 2.5, 5, 10, 30, and 50 μM) of each sample. Subsequently, the cells were incubated at 37 °C for an additional day, followed by two washings with PBS. US exposure was then administered using an Intelect^®^ MOBILE ULTRASOUND system (DJO, LLC) (0.5 W/cm^2^, 20% duty cycle, 1 MHz) for 1 min, after which a WST stock solution (100 μL) was added to each well. As negative controls, either 100 μL of the WST stock solution or an equivalent volume of distilled water was added to each well without the presence of any compound. Following a 4 h incubation period, the absorbance was recorded at 450 nm using a spectrophotometer (PowerWave XS, Bio-Tek, Winooski, VT, USA).

## 4. Conclusions

We developed a BODIPY-Zn complex-based sonosensitizer called **BSS-Zn** that incorporates a short hydrophilic PEG moiety. Comparative analyses of the Zn-free BODIPY-based system **BSS** and the well-known sensitizer ZnPc revealed that **BSS-Zn** exhibited more robust sonosensitizing activity under US irradiation. ROS generation assays employing three prevalent ROS (i.e., ^1^O_2_, •OH, and O_2_^•−^) indicators in solution and within MDA-MB-231 cells revealed that under US conditions, **BSS-Zn** generated not only ^1^O_2_ but also •OH and O_2_^•−^. **BBS-Zn** induced a significant SDT effect on MDA-MB-231 cells. **BSS-Zn** also demonstrated superior stability under physiological conditions, improved aqueous solubility due to the incorporation of a short hydrophilic PEG moiety, and potentially enhanced ROS generation under US irradiation. These advantages suggest that **BSS-Zn** offers improved bioavailability and therapeutic efficacy compared with conventional sonosensitizers (e.g., ZnPc). Although this study primarily focused on in vitro evaluations, future research will investigate the biodistribution, stability, tumor-targeting ability, and therapeutic efficacy of **BSS-Zn** in vivo to further establish its clinical potential as a promising sonosensitizer for SDT. This study not only confirms the potential of utilizing BODIPY—a well-known photosensitizer—as a sonosensitizer but also serves as a valuable reference for developing innovative sonosensitizers through Zn complexation.

## Data Availability

Data are available on request.

## Supplementary Materials

The following supporting information can be downloaded at: https://www.mdpi.com/article/10.3390/molecules30071587/s1, Figure S1: MALDI-TOF/TOF-MS spectrum of **BSS**; Figure S2: ^1^H NMR spectrum of **BSS** in DMSO-d_6_; Figure S3: ^13^C NMR spectrum of **BSS** in DMSO-d_6_; Figure S4: UV-Vis and fluorescence spectra of **BSS** (5.0 μM) in ethanol solution recorded at different concentrations of Zn^2+^ (0–1.0 equiv); Figure S5: Photosensitized ^1^O_2_ generation by **BSS-Zn** and **BSS**; Figure S6: Sonosensitized ROS generation; Figure S7: Sonosensitized ^1^O_2_ generation; Figure S8: Sonosensitized •OH generation; Figure S9: Sonosensitized O_2_^•−^ generation.
