# Supplementary material for: A Novel BODIPY-Zn Complex as Innovative Sonosensitizer for Enhanced Sonodynamic Therapy
Source: Molecules. 2025 Apr 2;30(7):1587. doi: 10.3390/molecules30071587 (PMC11990734; doi:10.3390/molecules30071587)
Supplement: Supplementary file 1 [file molecules-30-01587-s001.zip › molecules-3523407-supplementary.pdf]

Supporting Information

A Novel BODIPY-Zn Complex as Innovative Sonosensitizer for Enhanced Sonodynamic Therapy

Jungmin Lee [1,†], Soeun Lee [1,†], Gihoon Jo [1], Eunbin Hwang [1,2], Junhyoung Lee [1,2], Jiyou Han [1,*] and Hyo Sung Jung [1,*]

[1] Department of Biomedical & Chemical Sciences, Hyupsung University, Hwasung-si 18330, Republic of Korea
[2] Department of Gerontology (AgeTech-Service Convergence Major), Graduate School of East-West Medical Science, Kyung Hee University, Yongin-si 17104, Republic of Korea
* Correspondence: hanjiyou12@hanmail.net (J.H.), hs0101j@uhs.ac.kr (H.S.J.)
† These authors contributed equally to this work.

Table of Contents

Figure S1. MALDI-TOF/TOF-MS spectrum of **BSS**……………………………..……………...2
Figure S2. ^{1}H NMR spectrum of **BSS** in DMSO-d_6....……………………........……………….….2
Figure S3. ^{13}C NMR spectrum of **BSS** in DMSO-d_6....…………………….…...………………..…3
Figure S4. UV-Vis and fluorescence spectra of **BSS** recorded at different concentrations of Zn^{2+}……3
Figure S5. Photosensitized $^1O_2$ generation by **BSS-Zn** and **BSS**……………………………….....4
Figure S6. Sono-sensitized ROS generation.......……………………………..…………………5
Figure S7. Sono-sensitized $^1O_2$ generation....…………………………………...………….……5
Figure S8. Sono-sensitized •OH generation....………………………………..…………………6
Figure S9. Sono-sensitized $O_2^{•-}$ generation…………...…………………..……………….………6

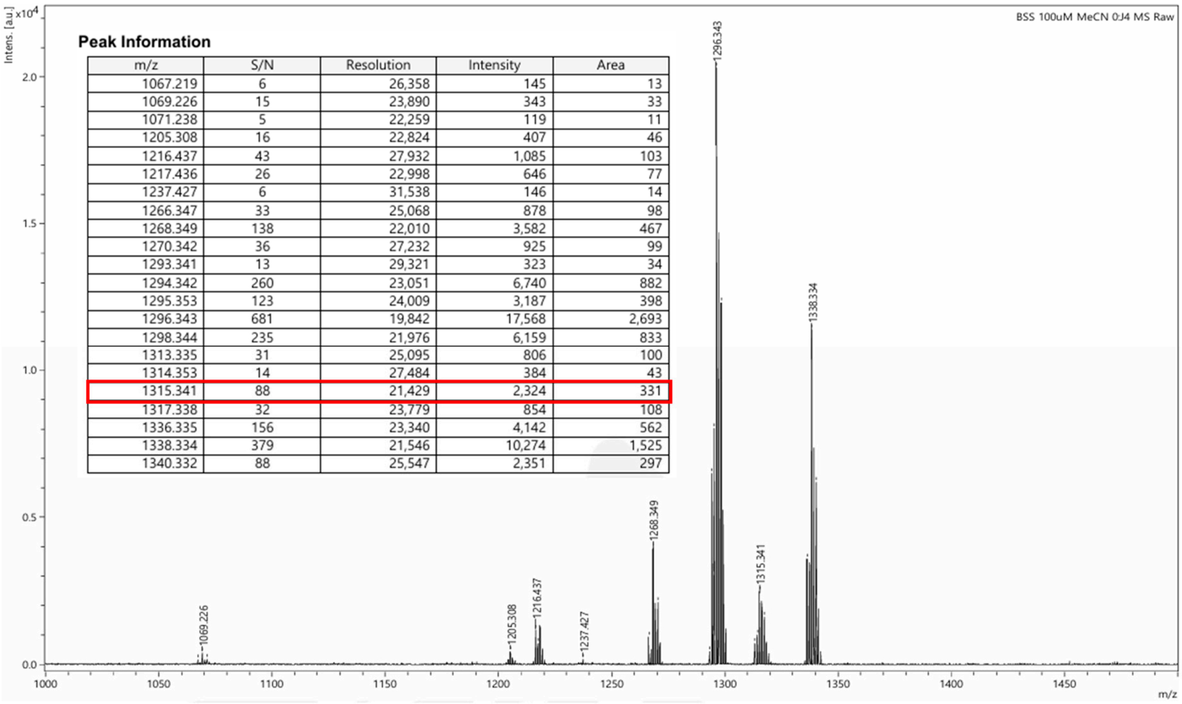

Peak Information

m/z	S/N	Resolution	Intensity	Area
1067.219	6	26,358	145	13
1069.226	15	23,890	343	33
1071.238	5	22,259	119	11
1205.308	16	22,824	407	46
1216.437	43	27,932	1,085	103
1217.436	26	22,998	646	77
1237.427	6	31,538	146	14
1266.347	33	25,068	878	98
1268.349	138	22,010	3,582	467
1270.342	36	27,232	925	99
1293.341	13	29,321	323	34
1294.342	260	23,051	6,740	882
1295.353	123	24,009	3,187	398
1296.343	681	19,842	17,568	2,693
1298.344	235	21,976	6,159	833
1313.335	31	25,095	806	100
1314.353	14	27,484	384	43
1315.341	88	21,429	2,324	331
1317.338	32	23,779	854	108
1336.335	156	23,340	4,142	562
1338.334	379	21,546	10,274	1,525
1340.332	88	25,547	2,351	297

Figure S1. MALDI-TOF/TOF-MS spectrum of **BSS**.

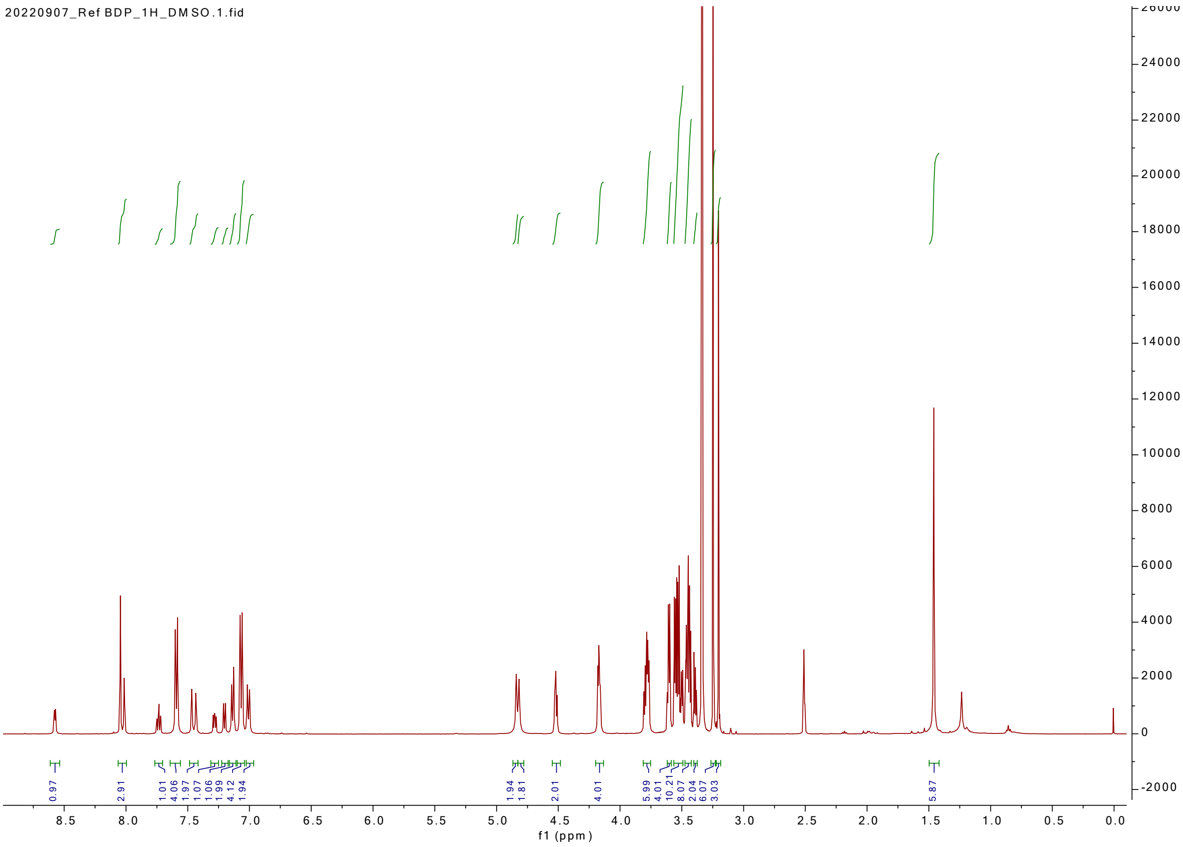

Figure S2. ^{1}H NMR spectrum of **BSS** in DMSO-d_6.

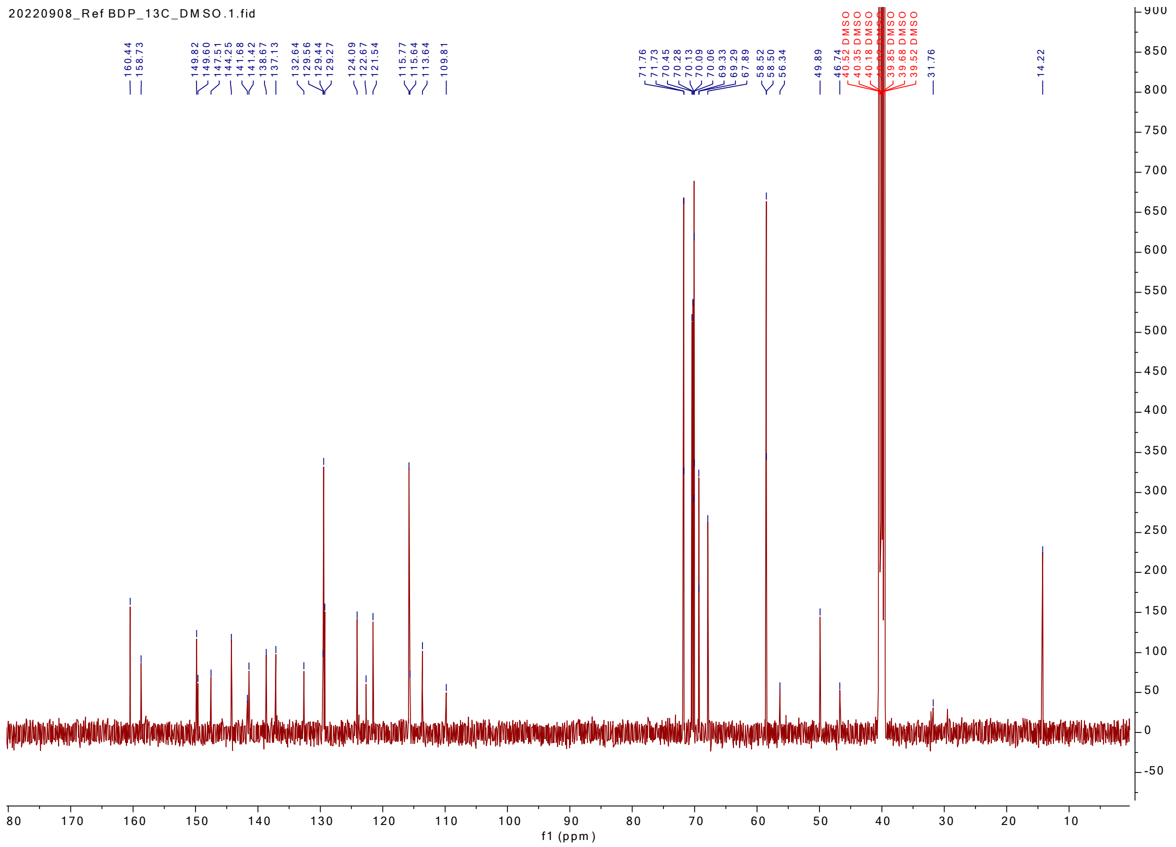

Figure S3. ^{13}C NMR spectrum of **BSS** in DMSO-d_6.

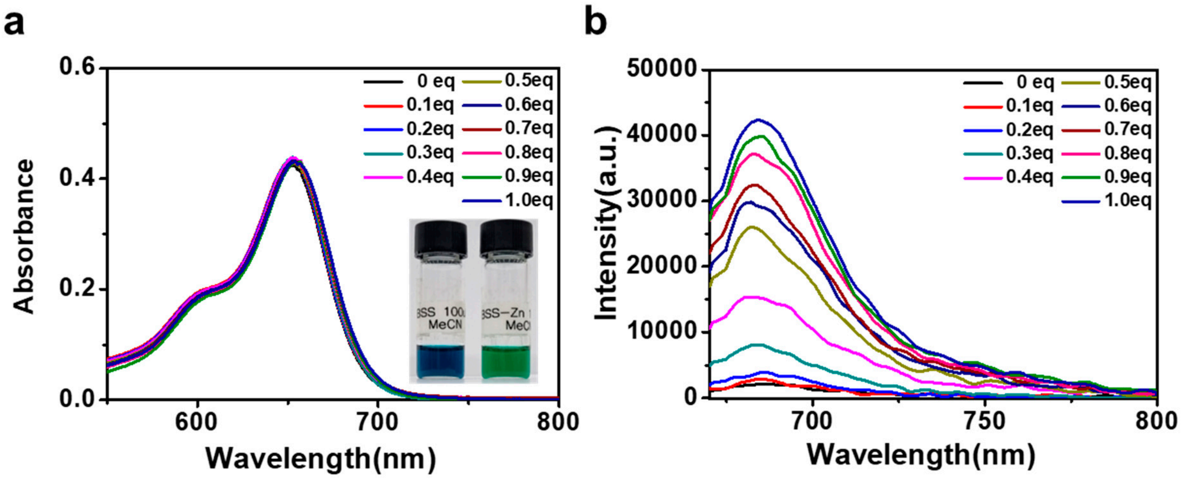

Figure S4. (a) UV-Vis and (b) fluorescence spectra of **BSS** (5.0 μM) in ethanol solution recorded at different concentrations of Zn^{2+} (ranging from 0 to 1.0 equiv.). Excitation at 660 nm. (slit = 2.5/2.5).

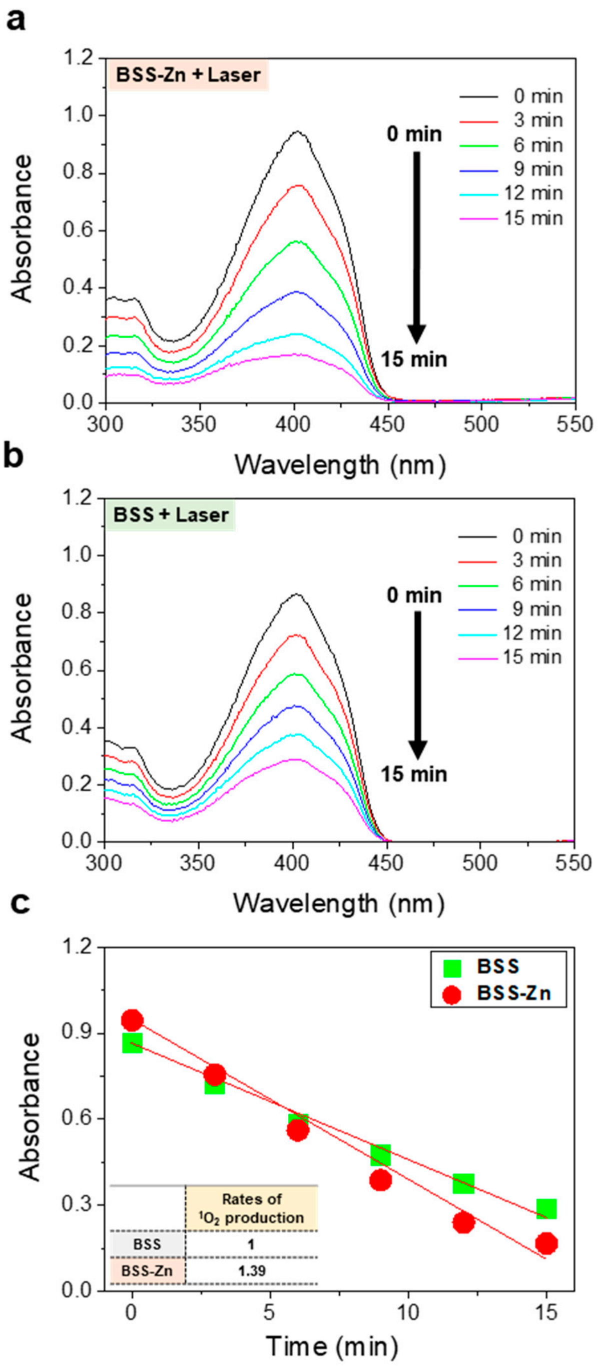

Figure S5. Photosensitized $^1O_2$ generation by **BSS-Zn** and **BSS**. Time-dependent absorption spectral changes seen for 40 µM solutions of 1,3-diphenylisobenzofuran (DPBF) containing 1 µM of (a) **BSS-Zn** or (b) **BSS**. Excitation at 660 nm (slit width = 15-1.5, Xe-lamp). (c) Plots of the change in the absorption intensity at 412 nm for the experiments shown in (a-b). The rates of $^1O_2$ production are represented by the relative slope between **BSS-Zn** and **BSS** shown on the graph.

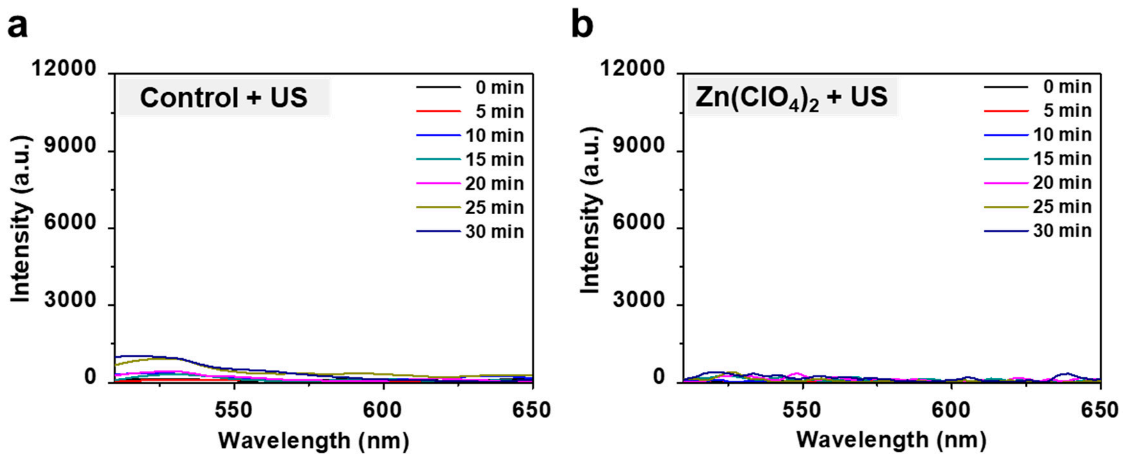

Figure S6. Sono-sensitized ROS generation. Time-dependent emission spectral changes seen for solutions of 10 μM dichlorodihydrofluorescein (DCF) containing (a) no sensitizer or (b) 5 μM $Zn(ClO_4)_2$ ions (as the perchlorate salt) under US exposure (0.5 W/cm^2, 20% duty cycle, 1 MHz); irradiation was effected at 500 nm (slit width = 1-2.5, Xe-lamp) in the two experiments.

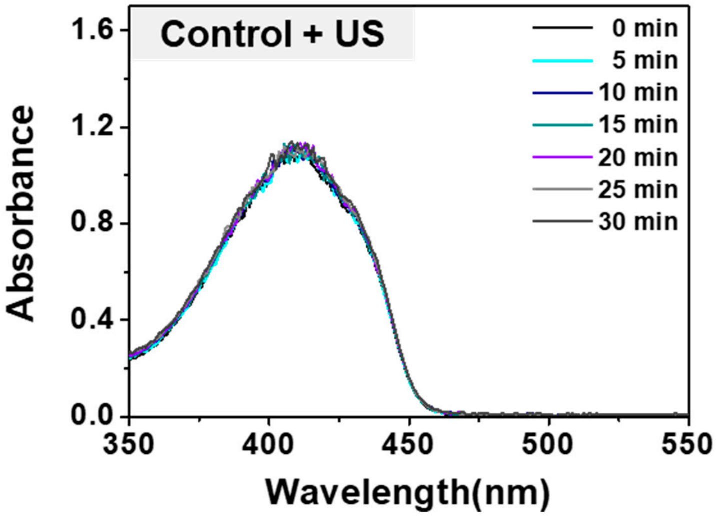

Figure S7. Sono-sensitized $^1O_2$ generation. Time-dependent absorption spectral changes seen for solutions of 10 μM DPBF under US exposure (0.5 W/cm^2, 20% duty cycle, 1 MHz).

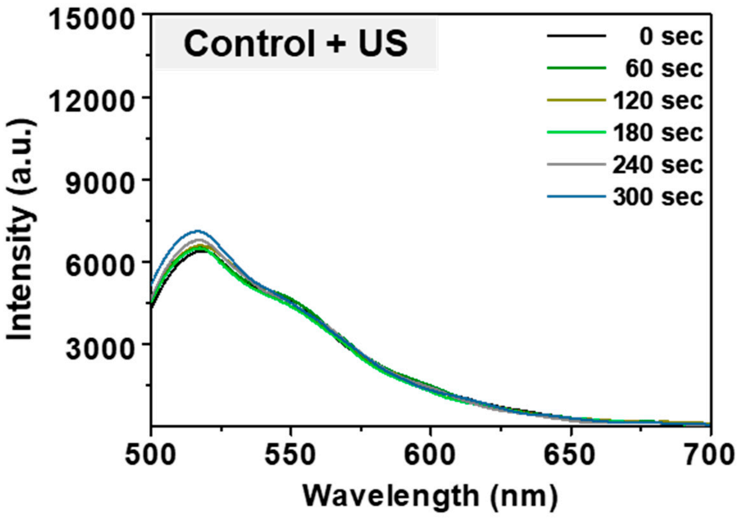

Figure S8. Sono-sensitized •OH generation. Time-dependent emission spectral changes seen for solutions of 10 μM HPF under US exposure (0.5 W/cm^2, 20% duty cycle, 1 MHz); irradiation was effected at 490 nm (slit width = 5-5, Xe-lamp).

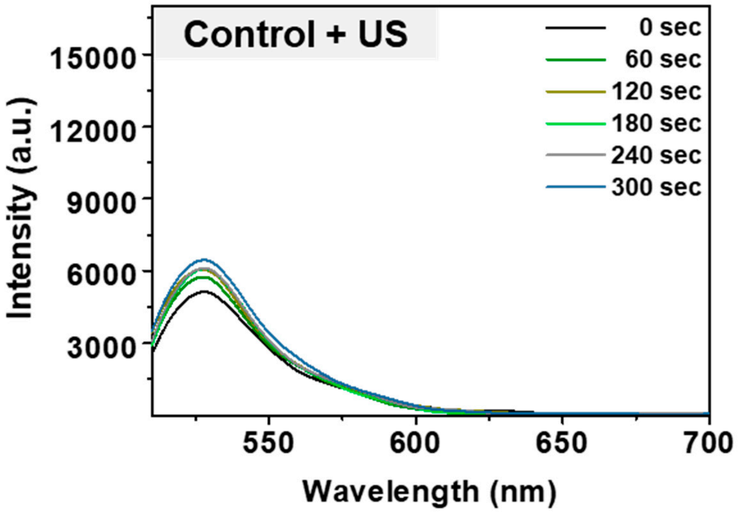

Figure S9. Sono-sensitized $O_2^{\bullet -}$ generation. Time-dependent emission spectral changes seen for solutions of 10 μM DHR123 under US exposure (0.5 W/cm^2, 20% duty cycle, 1 MHz); irradiation was effected at 500 nm (slit width = 2.5-5, Xe-lamp).
